# Autologous stem cell transplantation in tandem with Anti-CD30 CAR T-cell infusion in relapsed/refractory CD30^+^ lymphoma

**DOI:** 10.1186/s40164-022-00323-9

**Published:** 2022-10-17

**Authors:** Peiling Zhang, Xiuxiu Yang, Yang Cao, Jue Wang, Mi Zhou, Liting Chen, Jia Wei, Zekai Mao, Di Wang, Yi Xiao, Haichuan Zhu, Shangkun Zhang, Tongcun Zhang, Yicheng Zhang, Jianfeng Zhou, Liang Huang

**Affiliations:** 1 Department of Hematology, Tongji Hospital, Tongji Medical College, Huazhong University of Science and Technology, 1095 Jiefang Avenue, Wuhan, 430030 Hubei China; 2 Immunotherapy Research Center for Hematologic Diseases of Hubei Province, Wuhan, 430030 Hubei China; 3 College of Life Science and Health, Wuhan University of Science and Technology, Wuhan, 430065 Hubei China; 4 Wuhan Bio-Raid Biotechnology CO., LTD, Wuhan, 430078 Hubei China

**Keywords:** Chimeric antigen receptor T cell therapy, Autologous hematopoietic stem cell transplantation, CD30, Hodgkin lymphoma, Anaplastic large cell lymphoma, Refractory or relapsed lymphoma

## Abstract

**Background:**

Long-term outcome is unfavourable for relapsed/refractory (r/r) lymphoma patients who are resistant to salvage chemotherapy, even after subsequent autologous stem-cell transplantation (ASCT). Although anti-CD30 chimeric antigen receptor (CAR30) T-cell therapy induces high response rates in these patients, the duration of response is relatively limited.

**Methods:**

This open-label, single-center and single-arm pilot study investigated the safety and efficacy of ASCT in tandem with CAR30 T-cell infusion in r/r CD30^+^ lymphoma. The primary endpoint was safety and key secondary endpoint was overall response rate, overall survival, progression-free survival, and duration of response.

**Results:**

Five classical Hodgkin lymphoma (cHL) patients and 1 anaplastic lymphoma kinase (ALK)-negative anaplastic large cell lymphoma (ALCL) patient were enrolled. The median age was 24 years. No patient had prior ASCT. Three patients (50.0%) relapsed for ≥ 2 times and 3 patients (50.0%) had primary refractory diseases. All had a Deauville score of 4 or 5, and 5 patients (83.3%) had a stable or progressive disease (SD/PD) at enrollment. All patients received myeloablative chemotherapy and infused CD34-positive hematopoietic stem cells (HSCs) and CAR30 T cells in tandem, with a median dose of 3.9 × 10^6^/kg and 7.6 × 10^6^/kg, respectively. Five paitents presented with cytokine release syndrome (CRS), all of which were grade 1. No neurotoxicity was observed. All patients had successful HSCs engraftment and reached an objective response, including 5 (4 cHL and 1 ALCL, 83.3%) with a complete response (CR) and 1 with a partial response (PR). With a median follow-up of 20.4 (range, 12.1–34.4) months, all remained alive and maintained their responses.

**Conclusion:**

Our work demonstrates the combined administration of ASCT and CAR30 T-cell therapy is well-tolerate and highly effective in r/r cHL and ALCL, even in PET-positive or chemorefractory patients who are expected to have inferior outcome after ASCT, although further large-scaled validation in prospective clinical trial is warranted.

*Trial registration* The trial was registered with the Chinese Clinical Trial Registry (ChiCTR, number ChiCTR2100053662).

**Supplementary Information:**

The online version contains supplementary material available at 10.1186/s40164-022-00323-9.

## Background

Approximately 80% of patients with classical Hodgkin lymphoma (cHL) are treated with first-line therapy [1]. Second-line high-dose chemotherapy (HDT) or targeted chemotherapy followed by autologous stem cell transplantation (ASCT) is recommended in patients with relapsed/refractory (r/r) cHL [2]. However, durable remission after HDT/ASCT was achieved in only approximately half of the patients. Long-term survival is most likely achieved in patients who are sensitive to salvage therapy and have a complete response (CR) before ASCT, whereas outcomes are unfavorable in patients with residual disease. Currently, positron emission tomography (PET)-adapted approaches are widely used to treat cHL. Patients with a negative [18F] fluorodeoxyglucose (FDG) activity determined by PET (Deauville 1–3) before ASCT had an event-free survival (EFS) of 80.0% *versus* 28.6% for patients with a positive result (Deauville 4–5) [3]. With the goal of achieving a PET-negative CR, patients with active disease are candidates for alternative salvage chemotherapy, radiotherapy, CD30-directed therapy, checkpoint inhibitors, or participation in clinical trials.

The limited expression of CD30 in normal tissues and its consistent overexpression in cHL and anaplastic large cell lymphoma (ALCL) have encouraged the development of CD30-directed therapy [4]. Brentuximab vedotin (BV), a CD30-directed antibody–drug conjugate, has been approved for the treatment of cHL and peripheral T-cell lymphoma and was commercially available in China in 2020. Currently, clinical trials of anti-CD30 chimeric antigen receptor (CAR30) T-cell therapy are actively conducted in r/r cHL and ALCL, with an overall response rate (ORR) of 53.0–78.0% and a limited median progression-free survival (PFS) of approximately 6.0 months [5–7], indicating the need for novel strategies to further improve its long-term disease control. In our previous study on CAR30 T-cell therapy followed by anti-PD-1 antibody maintenance in r/r cHL and ALCL, the best CR rate was 77.8% with a median PFS of 13.0 months [5]. Owing to the potential synergistic effects of the combined administration of myeloablative ASCT and adoptive T-cell immunotherapy [8], we conducted an open-label, single-center, single-arm pilot study to explore the safety and efficacy of ASCT in tandem with CAR30 T-cell infusion in r/r CD30^+^ lymphoma.

## Methods

### Study design and enrollment

We carried out an open-label, single-center and single-arm pilot study to evaluate the safety and efficacy of tandem ASCT and CAR30 T cell infusion in r/r CD30 + lymphoma. This study was conducted in compliance with the principles of the Declaration of Helsinki. Approval from the Institutional Review Board of Tongji Hospital, Tongji Medical College, Huazhong University of Science and Technology, was obtained before the start of the study. Written informed consent was obtained from all participants. The primary endpoint was safety and the key secondary endpoint were ORR, overall survival (OS), PFS, and duration of response (DOR). This trial was registered in the Chinese Clinical Trial Registry (ChiCTR, number ChiCTR2100053662).

Eligible patients relapsed or refractory to at least two lines of salvage therapy. The diagnosis was verified according to the World Health Organization (WHO) classification of hematopoietic and lymphoid tissue tumors. CD30 expression was determined by immunohistochemical staining of malignant cells. The inclusion and exclusion criteria are outlined in the Supplemental Methods.

### Clinical procedures

**Fig. 1:**
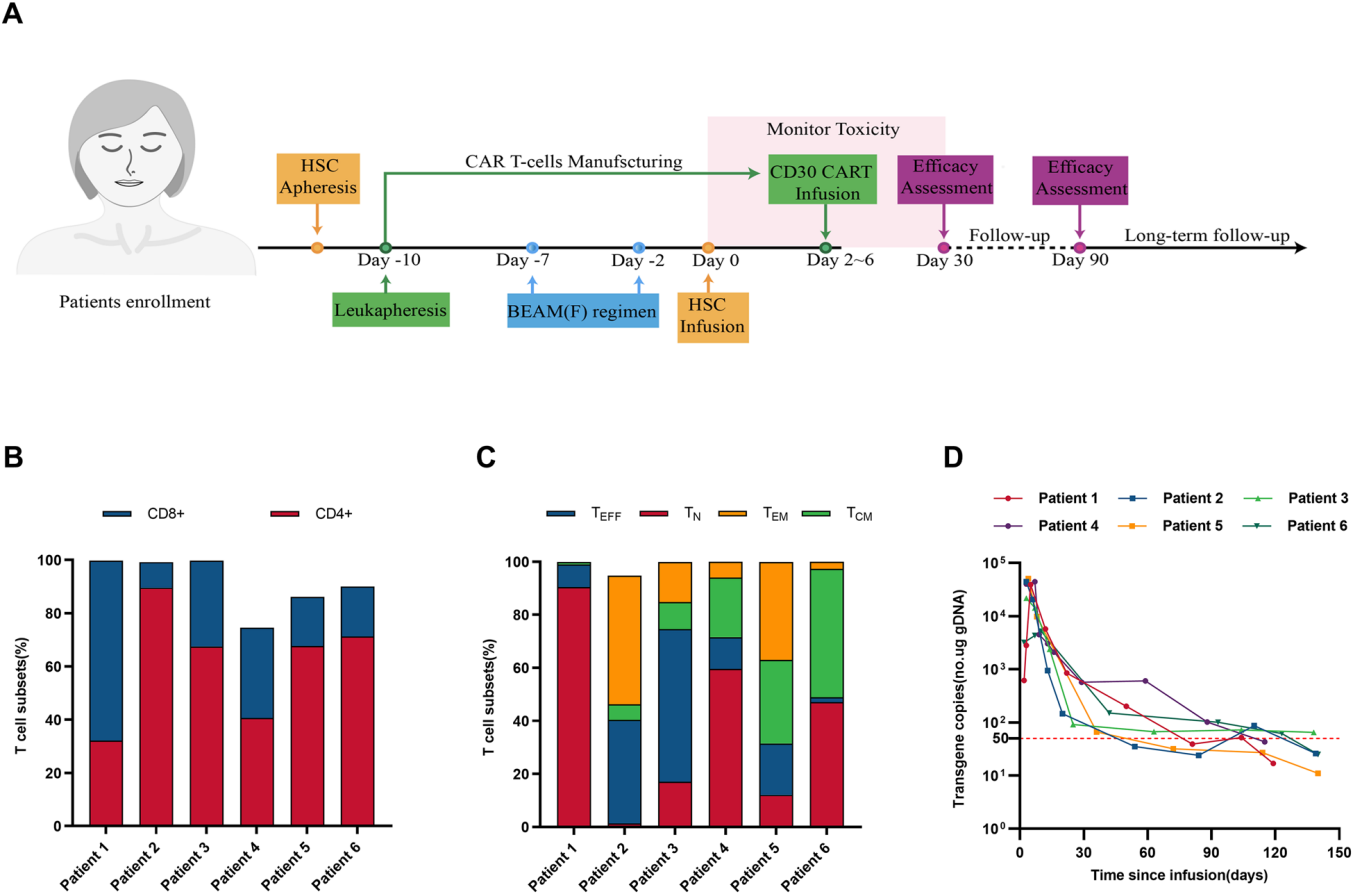
Flow chart, immunophenotype of CAR30 T-cell products and copies of CAR30 transgenes. **A**. Flow chart of study procedures. **B**. CD8/CD4 ratio of CAR30 T-cell products. **C**. The proportion of naïve T cells (T_N_, CD45RA + CCR7 +), effector (T_EFF_, CD45RA + CCR7-), central memory T cells (T_CM_, CD45RA-CCR7 +) and effector memory T cells (T_EM_, CD45RA-CCR7-) in the CAR30 T-cell products. **D**. The copies of CAR30 transgenes in the peripheral blood detected by ddPCR. The lower limit of quantitation was 50 copies/μg (the horizontal red line)

Eligible patients received hematopoietic stem cells (HSCs) mobilization and apheresis for ASCT and lymphocyte apheresis to obtain adequate lymphocytes from peripheral blood for chimeric antigen receptor (CAR) T cell manufacturing. The CAR used in this trial is a third-generation CAR composed of a single-chain variable fragment (scFv) targeting CD30 (patent number: CN106589139B), two costimulatory domains from CD28 and 4-1BB, and a CD3ζ chain as the activation domain [5]. Validation of the CAR constructs and procedures for cell production and quality control assays have been described previously [5]. Assessment of T-cell subsets of CAR30 T-cell products was performed as previously described [9] before infusion. Patients will receive a standard dose of BEAM (300 mg/m^2^ bis-carmusitine, − 7 days; 200 mg/m^2^ etoposide, − 6 to − 3 days; 400 mg/m^2^ cytarabine, − 6 to − 3 days; and 140 mg/m^2^ melphalan, − 2 days) with or without fludarabine (25 mg/m^2^ for− 6 day) as preconditioning, which was determined at the discretion of the investigators based on the clinical conditions of patients. HSCs were infused on day zero, followed by infusion of CAR30 T cells on day 2 to day 6. Cryopreserved HSCs collected before enrollment were considered acceptable for infusion. The response was assessed by imaging evaluations every 3 months in the 1st year and every 6–12 months thereafter. All patients were followed-up until death, loss to follow-up, or withdrawal of consent. The clinical procedure is illustrated in Fig. 1A.

### Laboratory assessments and follow-up

The in vivo expansion of CAR30 T cells was detected using droplet digital polymerase chain reaction (ddPCR) [5]. The cytokine levels were measured according to the manufacturer’s instructions. The cytokine release syndrome (CRS) was evaluated according to the scale proposed by *Lee *et al. [10]. Immune effector cell-associated neurotoxicity syndrome (ICANS) and other adverse events (AEs) were graded according to the American Society for Transplantation and Cellular Therapy Consensus [10] and the National Cancer Institute Common Terminology Criteria for Adverse Events V5.0. Staging and responses were assessed according to the US National Comprehensive Cancer Network and Lugano Treatment Response Criteria. Disease stage and response to treatment were assessed using PET/CT or CT, and defined according to the National Comprehensive Cancer Network Guidelines and the Lugano 2014 Guidelines.

### Statistical analysis

PFS and OS were defined as the time from the date of the infusion of CAR30 T cell to the date of the first relapse or death due to any reason, respectively. DOR was estimated as the time from the first response to first progression or death. The Kaplan–Meier method was used to analyze the survival rates. Statistical significance was set at a two-sided *P*-value of < 0.05 (and two-sided) was assumed to be statistically significant. All statistical analyses were performed using GraphPad Prism version 8.

## Results

### Patients and baseline features

Between June 1, 2019, and May 1, 2021, six patients were enrolled in this study, including five with cHL and one with anaplastic lymphoma kinase (ALK)-negative ALCL. The median age of the patients was 24 years. None of the patients had a history of undergoing ASCT. Three patients (50.0%) relapsed ≥ 2 times, and three patients (50.0%) had primary refractory diseases. All had a Deauville score of 4 or 5, and 5 patients (83.3%) had a stable or progressive disease (SD/PD) to previous treatments at enrollment. None of the patients had previously received ASCT. At enrollment, 5 patients had SD/PD. The baseline characteristics of the enrolled patients are summarized in Table 1.

**Table 1:** Baseline characteristics of enrolled patients

Patient	Diagnosis and stage^*^	Age (years)	Gender	Extranodal involvements	IPI score	Previous treatments			Response assessment^†^ at enrollment	Deauville score^†^ at enrollment (5-point scale)	Preconditioning
						Lines of treatment	RT	Targeted theray			
P1	ALCL, IIIB	23	Male	No	2	2	No	Brentuximab	SD	4	BEAM
P2	NS HL, IIA	30	Male	Lung	1	4	Yes	Anti-PD-1 mAb	PD	5	BEAM
P3	MC HL, IVA	26	Female	No	3	6	Yes	Anti-PD-1 mAb, CAR30 T-cell therapy	PR	5	BEAM
P4	NS HL, IIA	18	Female	No	1	4	No	Anti-PD-1 mAb	PD	4	BEAMF
P5	NS HL, IVB	25	Male	Lung	2	7	Yes	Anti-PD-1 mAb	PD	5	BEAMF
P6	NS HL, IIIA	20	Male	Skin, muscles	2	4	Yes	Anti-PD-1 mAb	SD	5	BEAM

^*^According to Ann Arbor staging.

^†^According to Lugano criteria for response assessment (PET/CT-based) in lymphoma

*IPI* international prognostic index, *RT* radiation therapy, *ALCL* anaplastic large-cell lymphoma, *NS HL* nodular sclerosis Hodgkin lymphoma, *MC HL* mixed cellularity Hodgkin lymphoma, *mAb* monoclonal antibody, *SD* stable disease, *PD* progressive disease, *PR* partial remission, *BEAM* bis-carmusitine, etoposide, cytarabine and melphalan, *P* patient, *F* fludarabine

### Infusion and kinetics of CAR30 T-cells and ASCT

The clinical procedure is shown in the flowchart in Fig. 1A. All patients underwent HSCs mobilization, apheresis, and lymphocyte apheresis (Fig. 1A). The median manufacturing time of CAR30 T-cell was 14.0 (interquartile range [IQR], 13.3–14.0) days. The patients were administered BEAM for preconditioning, including two patients who received additional fludarabine treatment (Table 1). CD34^+^ cells were infused at day zero with a median dosage of 3.9 (IQR, 3.2–6.1) × 10^6^/kg and followed by the infusion of CAR30 T cells with a median dosage of 7.6 (IQR, 5.5–9.7) × 10^6^/kg at day 2 to day 6. The phenotype of CAR30 T cell products was detected before infusion, as shown in Fig. 1B, C. The CAR30 T-cell products of patient 2 showed a high proportion of CD4 and a low proportion of T_N_, which may suggest less persistence and efficacy [11]. The median copies of peak expansion of CAR30 T cells was 4.2 (IQR, 2.6–4.4) × 10^4^/ug DNA (Fig. 1D). Persistent CAR30 transgenes were detected in the peripheral blood with a median duration time of 3.8 (IQR, 3.5–4.0) months (Fig. 1D).

### Safety

**Fig. 2:**
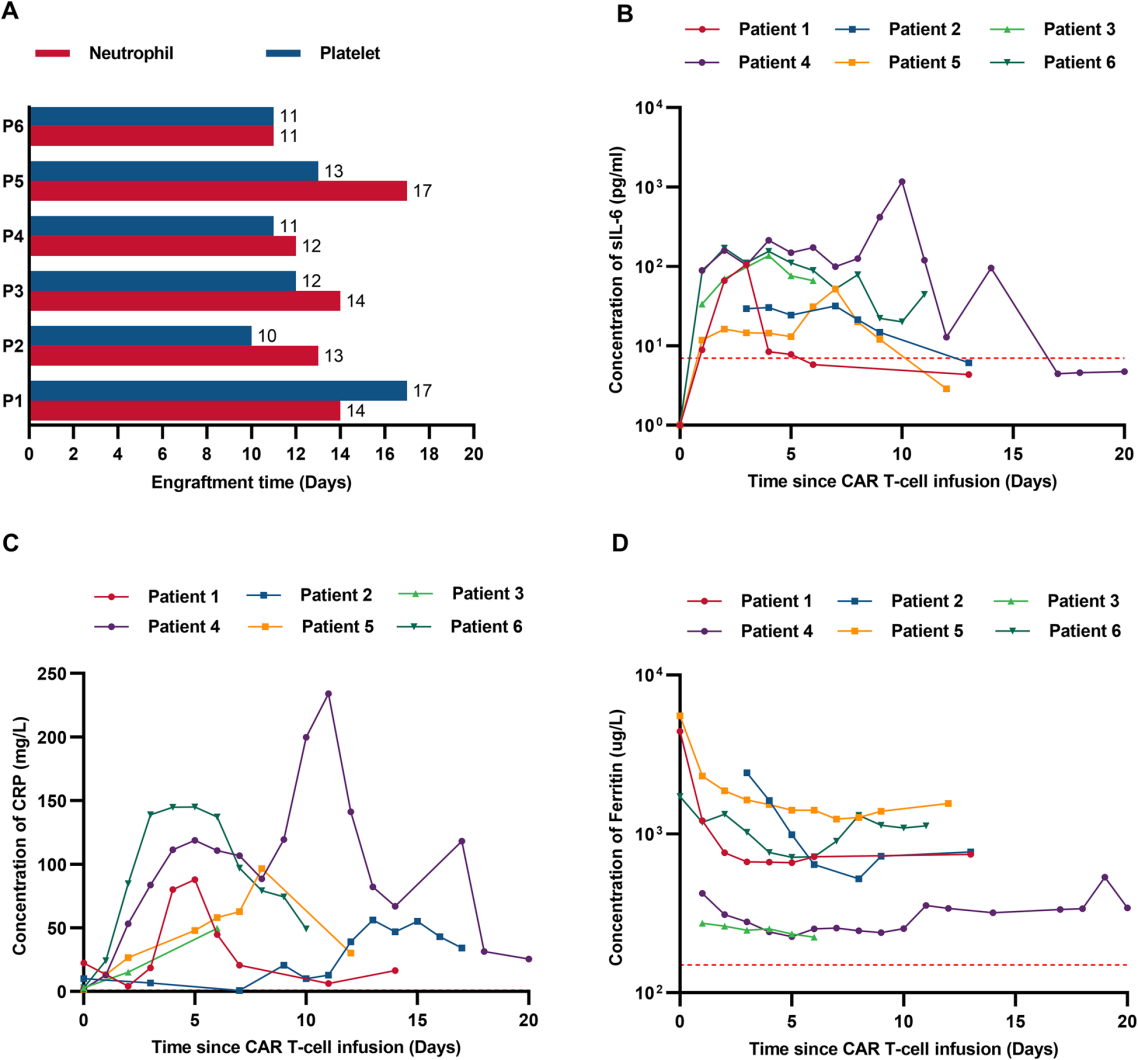
Multilineage engraftments and level of inflammatory cytokines of patients. **A**. Engraftment times of neutrophil or platelet of each patient after HSCs infusion. The red column represents the engraftment of neutrophil and the blue column represents the engraftment of platelet. **B**. The serum interleukin-6 (IL-6) level of each patient was assessed before and at serial time points after CAR T-cell infusion. **C**. The serum CRP level of each patient was assessed before and at serial time points after CAR T-cell infusion. **D**. The serum ferritin level of each patient was assessed before and at serial time points after CAR T-cell infusion. The horizontal red line denotes the normal limit of quantitation

AEs are listed in Table 2. Cytopenia was the most common severe AEs (grade ≥ 3) within the 1st month. All the patients had grade 3 neutropenia and thrombocytopenia. The engraftments of neutrophil or platelet occured at a median time of 13.5 (IQR,12.3–14.0) days or 11.5 (IQR, 11.0–12.8) days, respectively, suggesting rapid multilineage engraftments post ASCT (Fig. 2A). CRS developed in five (83.3%) patients at a median time of 3.0 (IQR, 3.0–4.0) days after chimeric antigen receptor (CAR) T-cell infusion (Additional file 1: Table S1), lagging behind the rise in interleukin-6 (Fig. 2B). All CRS were of grade 1 and mostly resolved within 7.0 (IQR, 2.0–7.0) days after supportive care. ICANS was not observed. Although preconditioned with BEAM, none of the patients experienced a severe infection within the 1st month. 8 months later, one (16.7%) of them developed mild pneumonia due to infection with *pneumocystis jirovecii* and *streptococcus* after withdrawal of co-trimoxazole prophylaxis, and recovered rapidly after antibiotic treatment. Infection is one of the most common AEs following CD19-directed CAR T-cell therapy, with incidence ranging from 17.0% to 42.0% in the 1st month and 14.0–31.0% in the 1st year [12], suggesting the need for standardized infection prophylaxis [13].

**Table 2:** Grading of adverse event (AE) occurred in the 1st month^*^

AE	Grade of AE in each patient					
	P1	P2	P3	P4	P5	P6
CRS	1	1	1	1	0	1
ICANS	0	0	0	0	0	0
Secondary infection	0	0	0	0	0	0
Hypoxia	0	0	0	0	0	0
Pulmonary edema	0	0	0	0	0	0
Hypotension	0	0	0	0	0	2
Nausea	0	0	3	0	0	2
Vomiting	0	0	2	0	0	0
Diarrhea	0	2	2	2	0	2
Oral mucositis	0	2	0	0	0	0
Purpura	0	2	0	0	0	0
Neutropenia	3	3	3	3	3	3
Anemia	1	1	1	1	1	1
Thrombocytopenia	3	3	3	3	3	3
Prolonged APTT	0	0	1	0	0	1
Hyperuricemia	1	1	0	0	0	1
Transaminitis	1	1	0	1	1	1
Hypoalbuminemia	1	1	0	1	1	1

^*^Time calculated since CAR T-cell infusion

*P* patient, *CRS* cytokine release syndrome, *ICANS* immune effector cell associated neurotoxicity syndrome, *APTT* activated partial thromboplastin time

### Efficacy and long-term follow-up

**Fig. 3:**
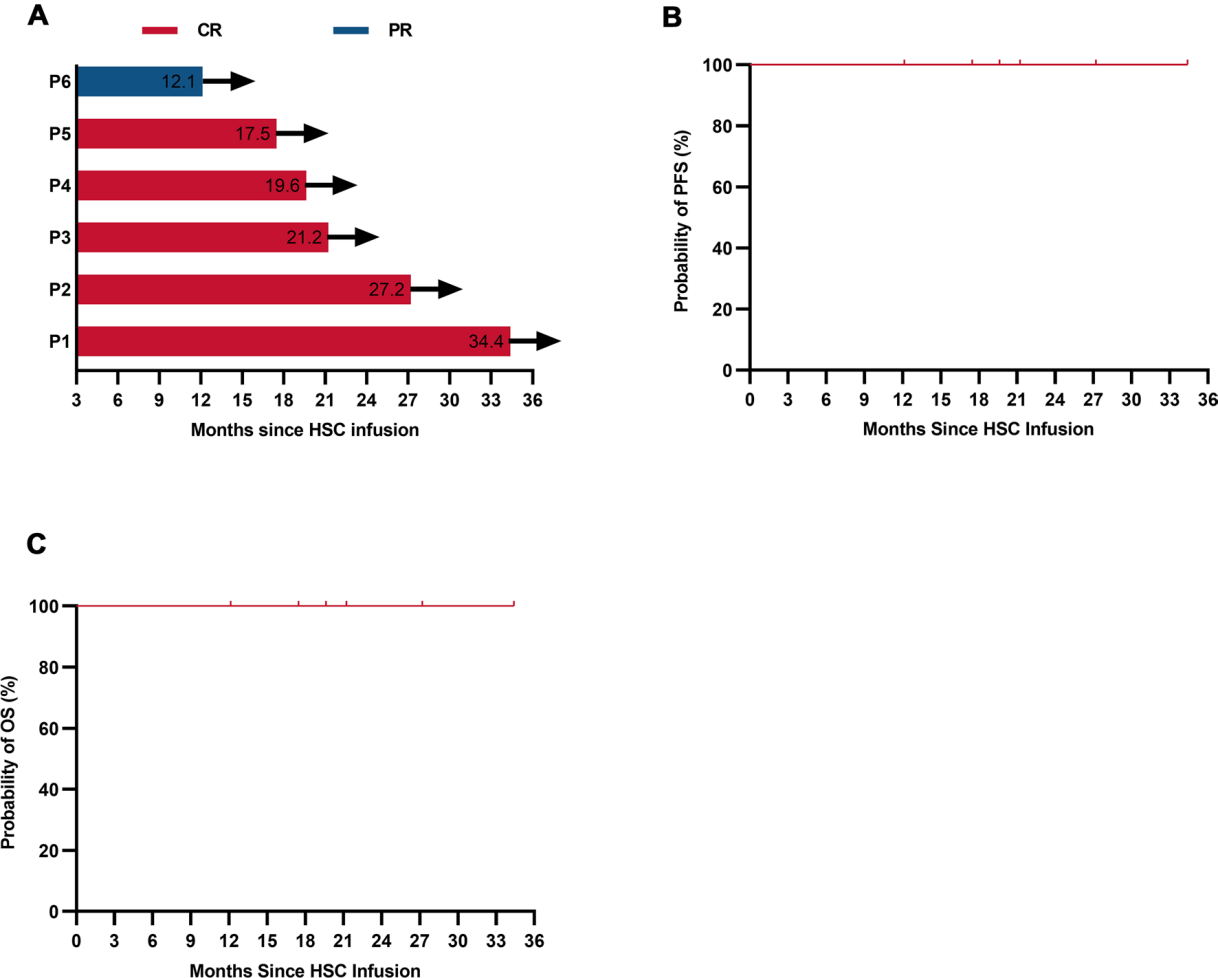
Long-term outcome of patients. **A**. Duration time of remission after ASCT following CAR30 T-Cell infusion. The red column represents complete response (CR) and the blue column represents partial response (PR). Arrow indicates ongoing response. **B**, **C**. Propability of PFS (B) or OS (C) of enrolled patients after ASCT following CAR30 T-Cell infusion. Tick marks represent censored data. *P* patient

At month 3 post-HSCs infusion, all patients achieved objective responses, including five (83.3%) with a CR and one (16.7%) with a patial response (PR) (Fig. 3A). With a median follow-up of 20.4 (range, 12.1–34.4) months, the median PFS and OS were not reached (Fig. 3B, C). On January 31, 2022, the data cutoff date, all patients maintained their responses and remained alive without disease relapse or progression (Fig. 3A), indicating that superior response and durable remission could be achieved when ASCT and CAR T-cell infusion were sequentially administered. Of note, CR was sustained in all five (83.3%) patients who had an SD/PD at enrollment, which highlighted that even in PET-positive or chemorefractory patients who were expected to have inferior outcomes after ASCT, long-term disease control could be realized when ASCT followed by CAR T-cell infusion.

## Discussion

Previous studies have demonstrated up to 30% of patients with r/r CD30^+^ lymphoma will not be ineligible for ASCT because of the lack of chemosensitivity, which have an extremely poor prognosis [14]. Even in approximately 50% of patients eligible for ASCT, recurrence occurs post ASCT, which partially due to less than a CR to second-line therapy before ASCT [15]. For ASCT, a remission status of PR and above is necessary. Patients with FDG-PET positivity after HDT showed poor outcomes [3]. Therefore, new treatment options are urgently needed. CD30 is often expressed in cHL and ALCL and has proven to be an excellent target for immune-based therapies [16]. In CD30^+^ lymphoma, the antibody–drug conjugate against CD30, BV has shown impressive activity, but the remissions are sustained only in less than 25% of patients with r/r lymphoma [17]. Although previous clinical trials of CAR30 T-cell therapy provided preliminary efficacy in r/r CD30^+^ lymphoma, a considerable number of patients failed to achieve durable CR. Carlos et al. reported the outcomes of 41 patients who received CAR30 T-cell. The complete remission rate for all evaluable patients was 60.0%, and 1-year PFS is 36.0% [7]. Wang et al. reported that out of 18 patients who received CAR30 T-cells, the remission rate for PR and above was 67.0%, with a median PFS of 6.0 months [18]. Timothy J et al. showed that the CR rate of patients receiving CAR30 T-cells was 67.0%, with a median PFS of 11 months [6]. Furthermore, our previous study have shown that the CR rate of patients received CAR30 T-cells followed by anti-PD-1 antibody maintenance was 77.8% in total of nine patients [5], with updated 2-year PFS of 44.4% with a median follow-up of 38.3 months (range, 0–63 months) (Additional file 1: Figure S1), indicating novel strategies to improve the efficacy of CAR30 T-cells were needed.

Accumulating evidence suggests that the efficacy of adoptive T-cell therapy can be enhanced by myeloablative stem cell transplantation for the treatment of B-cell lymphoma [8, 19]. Myeloablative preconditioning administered before adoptive T-cell infusion eliminates tumor cells and immunosuppressive elements in the microenvironment and induces a favorable cytokine profile to recruit and motivate immune cells, which may improve the efficacy of CAR T-cell therapy [20]. In addition, reduced tumor burden and suppressed monocytes and macrophages in the tumor microenvironment may alleviate the severity of CRS and ICANS [21]. Thus, this combination may result in enhanced synergistic response, mild toxicity, reduced relapse, and durable remission. In our study, when treated with ASCT in tandem with CAR30 T-cell infusion, superior response and survival can be obtained, even in 5 patients with a SD/PD (83.3%) before transplantation, for whom ASCT alone can hardly provide durable disease. We report our results in six r/r CD30^+^ lymphoma patients (five with cHL and one with ALCL), with ORR and CR rates of 100% and 83.3%, respectively. The median OS and PFS of the patients were not reached with a median follow-up of 20.4 months, which was far superior to that of the same CAR30 T cell infusion without ASCT [5] (Additional file 1: Figure S1). The therapy was well-tolerated, with only grade 1 CRS and no CRES. In contrast to our previous study of CAR30 T-cell therapy [5], the peak of IL-6 and serum ferritin levels during CRS were slightly lower in patients treated with ASCT in tandem with CAR30 T-cell infusion, while the expansion of CAR-T cells was no significant difference, although further large-scale validation in double-arm clinical trials is warranted. Tolerable CRS and excellent PFS suggest that potential synergistic effects may be generated when ASCT and CAR T cell therapies are combined. In addition, a second infusion of CAR19 T-cells has been considered a strategy to overcome CAR19 T-cell therapy failure in r/r B-cell malignancies [22]. In our study, patient 3 received mouse-derived CAR30 T-cell treatment prior to ASCT in tandem with CAR30 T-cell infusion, and ddPCR showed durable persistence of CAR30 T-cells, revealing that ASCT in tandem with CAR30 T-cell infusion may improve the efficacy of repeated CAR-T cell infusions.

The limitations of this study include the fact that only one patient received BV treatment in this study because of the approval of BV from the National Medical Products Administration in May 2020, later than the beginning of this study. However, all other patients received anti-PD-1 antibody treatment, which showed better clinical efficacy than that of BV [23].

## Conclusions

Taken together, tandam administration in ASCT and CAR T-cell therapy is well tolerated and highly active in r/r cHL and ALCL, although further large-scale validation in prospective clinical trials is warranted.

## Supplementary Information

**Additional file 1: **Supplemental Materials.

## Data Availability

The datasets used and analysed during the current study are available from the corresponding author on reasonable request.

## Abbreviations

AEs: Adverse events
ALCL: Anaplastic large cell lymphoma
ALK: Anaplastic lymphoma kinase
ASCT: Autologous stem-cell transplantation
BV: Brentuximab vedotin
CAR: Chimeric antigen receptor
CAR30: Anti-CD30 chimeric antigen receptor
ChiCTR: Chinese clinical trial registry
cHL: Classical Hodgkin lymphoma
CR: Complete response
CRS: Cytokine release syndrome
ddPCR: Droplet digital polymerase chain reaction
DOR: Duration of response
EFS: Event-free survival
FDG: Fluorodeoxyglucose
HDT: High-dose chemotherapy
HSCs: Hematopoietic stem cells
HSCs: Hematopoietic stem cells
ICANS: Immune effector cell-associated neurotoxicity syndrome
IQR: Interquartile range
ORR: Overall response rate
OS: Overall survival
PET: Positron emission tomography
PFS: Progression-free survival
PR: Partial response
r/r: Relapsed/refractory
scFv: Single-chain variable fragment
SD/PD: Stable or progressive disease
WHO: World Health Organization
