# Supplementary material for: Autologous stem cell transplantation in tandem with Anti-CD30 CAR T-cell infusion in relapsed/refractory CD30+ lymphoma
Source: Exp Hematol Oncol. 2022 Oct 17;11:72. doi: 10.1186/s40164-022-00323-9 (PMC9578248; doi:10.1186/s40164-022-00323-9)
Supplement: Supplementary file 1 — Additional file 1: Supplemental Materials. [file 40164_2022_323_MOESM1_ESM.docx]

Supplement to ***Zhang et al. Autologous Stem Cell Transplantation***

***in Tandem with Anti-CD30 CAR T-cell Infusion***

***in Relapsed/Refractory CD30^+^ Lymphoma***

This Supplementary Information has been provided by the authors to give readers additional information about their work.

**CONTENTS**

[Supplemental Methods 3](#_Toc114149369)

[***Inclusion and exclusion criteria*** 3](#_Toc114149370)

[Supplemental Figures 5](#_Toc114149371)

[**Figure S1. Updated PFS of patients with CAR30 T-Cell infusion in our previous study.** 5](#_Toc114149372)

[Supplemental TABLEs 6](#_Toc114149373)

[**Table S1. Onset and resolution of CRS in each patient** 6](#_Toc114149374)

**Supplemental Methods**

***Inclusion and exclusion criteria***

**Inclusion criteria:**

Subjects must meet all the following criteria to be enrolled in this study:

1. Signed written informed consent obtained prior to any study procedures.
2. Age 18 to 70 years at the time of consent.
3. Subjects with CD30-positive lymphoma diagnosed by pathology and histology.
4. Relapsed or refractory CD30-positive lymphoma of the following histology: Hodgkin lymphoma (HL), anaplastic large cell lymphoma (ALCL) *et al*.
5. Eligible for ASCT and in one of the following conditions:
6. With relapsed or refractory disease after 2^nd^-line of salvage therapy.
7. Relapsed after CR1 and with high-risk clinical features, including early relapse (<12 months after completion of initial therapy), generalized systemic relapse, bulky disease (mediastinal disease greater than one-third of thoracic diameter, or mass greater than 10 cm), extranodal involvement *et al*.
8. With residual disease after at least 2 lines of treatment.
9. Subjects with measurable lesions.
10. Adequate organ function, defined as creatinine<2.5mg/dl; aspartate transaminase/alanine transaminase <3×upper limit of normal; SiO2 ≥ 95%; bilirubin < 2.0 mg/dl; LVEF > 40%.
11. Adequate vascular access for leukapheresis procedure.
12. Eastern Cooperative Oncology Group (ECOG) performance status of ≤ 2.
13. Estimated survival of ≥ 3 months.

**Exclusion criteria:**

Subjects who meet any of the following criteria will be excluded from participation in this study:

1. Pregnant or nursing women.
2. Planning pregnancy within 1 year.
3. Active hepatitis B, hepatitis C, or human immunodeficiency virus (HIV) infection at the time of screening.
4. Systemic fungal, bacterial, viral, or other infection that is not controlled, at the time of screening.
5. History of accepting systemic steroids treatments within 4 weeks.
6. Allergic to any cytokines or antibodies.
7. Participation in an investigational research study within 6 weeks before enrolled.
8. Present active graft-versus host disease.
9. History of mental disorders.
10. History of another primary malignancy.
11. Drug abuse and addiction.
12. Other conditions deemed inappropriate by the investigator for enrollment.

**Supplemental Figures**

**Figure S1. Updated PFS of patients with CAR30 T-Cell infusion in our previous study.**


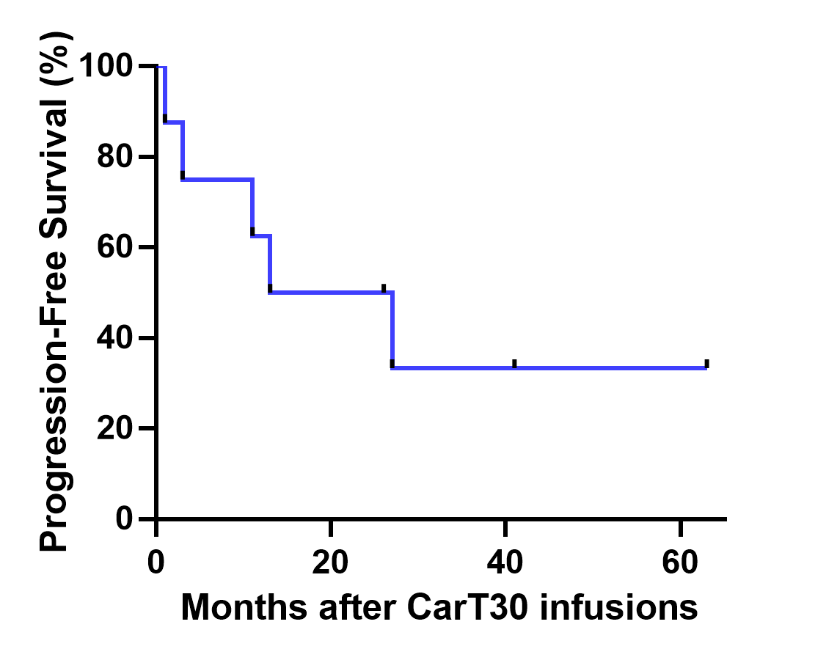


**Updated PFS of patients with CAR30 T-Cell infusion in our previous study.** Tick marks represent censored data.

**Supplemental TABLEs**

**Table S1. Onset and resolution of CRS in each patient**

| **Patient** | **Time to Onset^*^**  (Days) | **Grade** | **Intervention** | **Time to Resolution^†^**  (Days) |
| --- | --- | --- | --- | --- |
| P1 | 4 | 1 | Supportive | 2 |
| P2 | 1 | 1 | Supportive | 2 |
| P3 | 4 | 1 | Supportive | 2 |
| P4 | 3 | 1 | Supportive | 9 |
| P5 | - | 0 | - | - |
| P6 | 3 | 1 | Supportive | 7 |

^*^ Time calculated since CAR T-cell infusion. **^†^** Time calculated since onset of CRS.
